# ﻿*Oreocharispolyneura*, a new species of Gesneriaceae from southern Yunnan, China

**DOI:** 10.3897/phytokeys.214.93901

**Published:** 2022-11-22

**Authors:** Yan-Xiong Gong, Hong-Bo Ding, Xiang-Shuai Yan, Fang Wen, Yao-Hua Tian, Yun-Hong Tan

**Affiliations:** 1 Yunnan Institute of Tropical Crops, Jinghong, CN-666100, Yunnan, China Yunnan Institute of Tropical Crops Jinghong China; 2 Southeast Asia Biodiversity Research Institute, Chinese Academy of Sciences, Menglun, Mengla, CN-666303, Yunnan, China Southeast Asia Biodiversity Research Institute, Chinese Academy of Sciences Mengla China; 3 Center for Integrative Conservation, Xishuangbanna Tropical Botanical Garden, Chinese Academy of Sciences, Menglun, Mengla, CN-666303, Yunnan, China Xishuangbanna Tropical Botanical Garden, Chinese Academy of Sciences Mengla China; 4 Center of Conservation Biology, Core Botanical Gardens, Chinese Academy of Sciences, Menglun, Mengla, CN-666303, Yunnan, China Core Botanical Gardens, Chinese Academy of Sciences Mengla China; 5 Guangxi Key Laboratory of Plant Conservation and Restoration Ecology in Karst Terrain, Guangxi Institute of Botany, Guangxi Zhuang Autonomous Region and Chinese Academy of Sciences, Guilin, CN-541006, Guangxi, China Guangxi Institute of Botany, Guangxi Zhuang Autonomous Region and Chinese Academy of Sciences Guilin China; 6 Gesneriad Committee of China Wild Plant Conservation Association, National Gesneriaceae Germplasm Resources Bank of GXIB, Gesneriad Conservation Center of China, Guilin Botanical Garden, Chinese Academy of Sciences, Guilin, CN-541006, Guangxi, China Guilin Botanical Garden, Chinese Academy of Sciences Guilin China

**Keywords:** flora of Yunnan, morphology, new taxon, *Oreocharisrhytidophylla*, taxonomy

## Abstract

*Oreocharispolyneura*, a new species from southern Yunnan, China, is described and illustrated. It is morphologically similar to *O.rhytidophylla* by having more obvious lateral veins forming a crosslinked network on the adaxial surface of leaf blades, but can be distinguished by having more lateral veins (12–15 pairs vs. 7–9 pairs) of leaf blades, shorter corolla tubes (the length ratio of corolla tube to corolla lobes = 1.2–2.9 vs. 3.4–6) and shorter pistils (6–8 mm long vs. 27–30 mm long). In addition, a detailed morphological description, a photographic illustration, the distribution and phenology of the new species are presented.

## ﻿Introduction

In recent years, the genus *Oreocharis* Benth. has been a hotspot for the research of the family Gesneriaceae. The newly circumscribed genus *Oreocharis* is recently enlarged by incorporating ten other genera (*Ancylostemon* Craib, *Bournea* Oliv., *Dayaoshania* W.T.Wang, *Deinocheilos* W.T.Wang, *Isometrum* Craib, *Opithandra* B.L.Burtt, *Paraisometrum* W.T.Wang, *Thamnocharis* W.T.Wang, *Tremacron* Craib, *Briggsia* Craib s.str.) with high floral diversity (Möller et al. 2011; Weber et al. 2013), although *Bournea* was later reinstated as an independent genus (Chen et al. 2020a). Hitherto, *Oreocharis* s.l. contains ca. 150 taxa mainly distributed in southern and southwestern China (Wen et al. 2021), with a few of them (ca. 15 species) expanding to Bhutan, India, Japan, Myanmar, Thailand, and northern and central Vietnam (Möller et al. 2016, 2017, 2018; Cai et al. 2020a; Le et al. 2022). In China, southern Yunnan is rich in species diversity of the genus, with about 38 species recorded in this area (Li and Wang 2004; Möller et al. 2011; Chen et al. 2012, 2013, 2020b; Tan et al. 2013, 2015; Rossini and Freitas 2014; Cai et al. 2015, 2020; Cai and Dao 2020).

**Figure 1.**
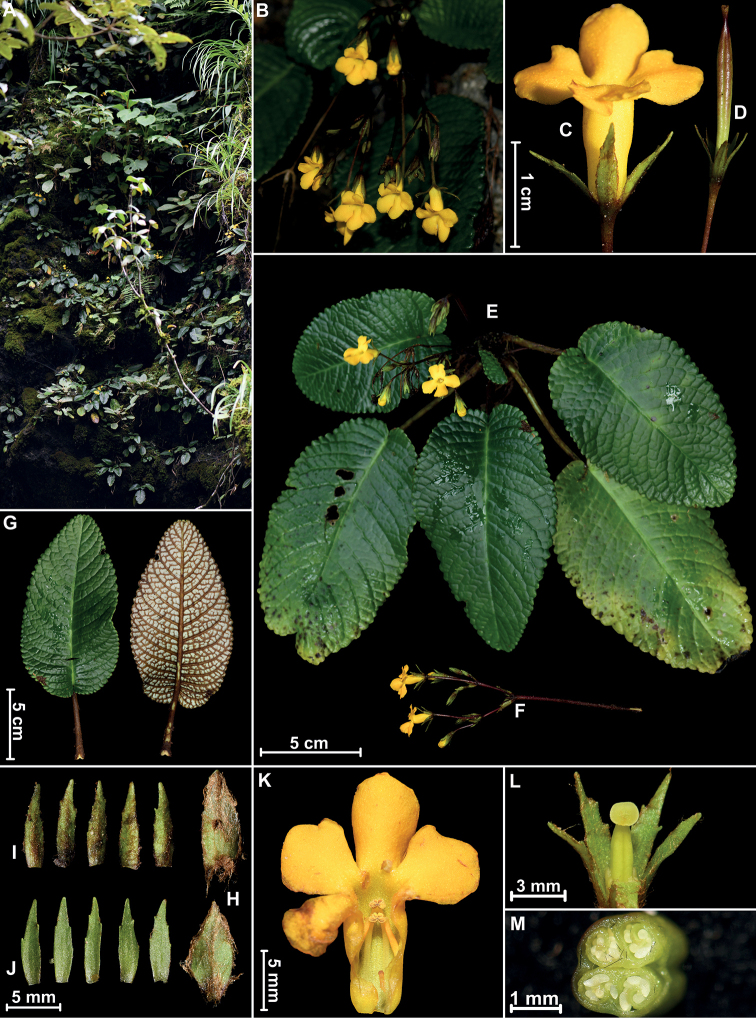
*Oreocharispolyneura* Y.H.Tan, F.Wen & Y.X.Gong, sp. nov. **A** habit **B** inflorescence **C** behind view of flower **D** fruit **E** whole plant **F** inflorescence with scale **G** adaxial and abaxial leaf surfaces **H** bracts **I, J** calyces **K** opened corolla showing stamens and staminode **L** pistil with disc and calyces **M** cross section of fruit. Photographed by Yan-Xiong Gong.

**Figure 2.**
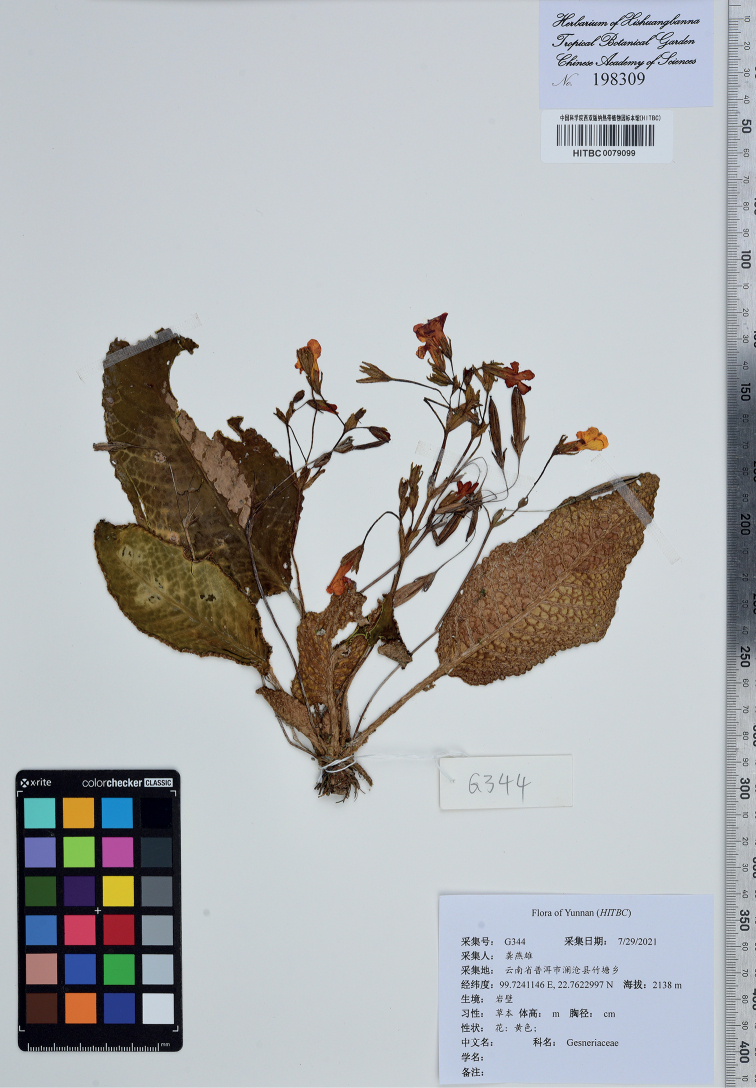
Type sheet of *Oreocharispolyneura* Y.H.Tan, F.Wen & Y.X.Gong, sp. nov.

**Figure 3.**
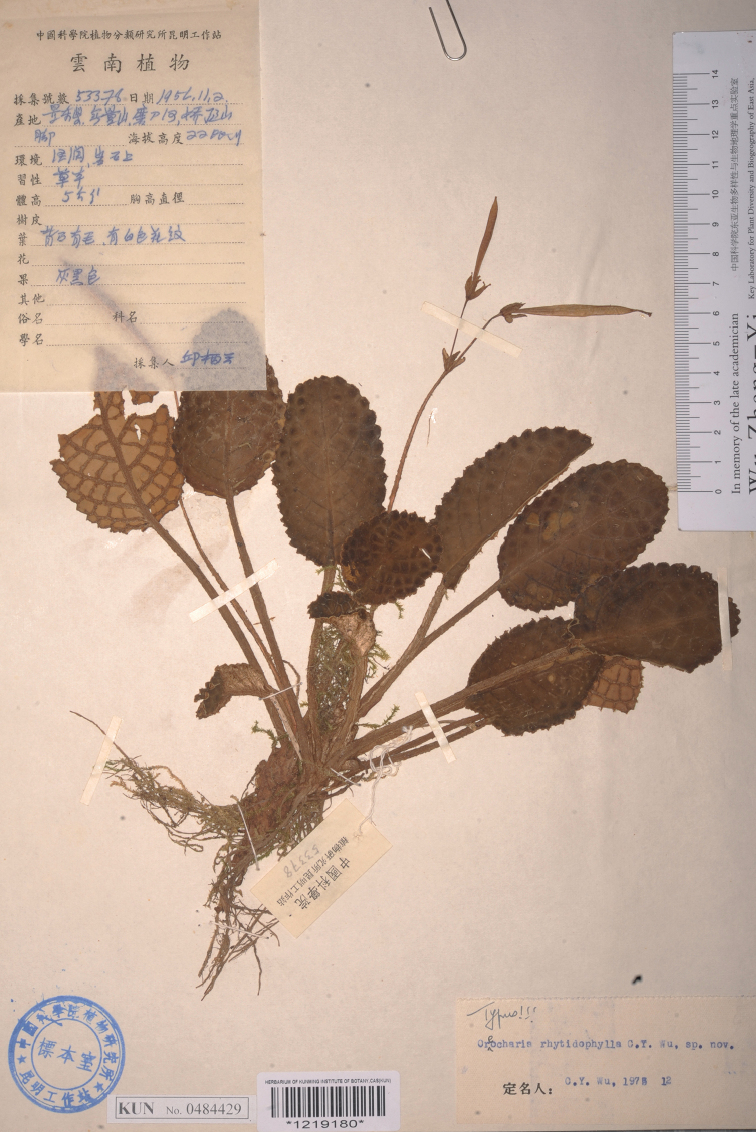
Type sheet of *Oreocharisrhytidophylla* C.Y. Wu ex H.W. Li.

In 2021, during the expedition of the plant resources of tropical Yunnan in China, we collected an interesting plant of Gesneriaceae from Lancang county at anthesis (Figs 1, 2). Judging from the vegetative habit and floral characteristics, we considered it a member of *Oreocharis*. Upon careful comparisons of diagnostic morphological and anatomical features from the closely related species from China and adjacent regions, we determined that the plant is new to science and thus describe it herein. Its morphological characters are compared with the closely related species *Oreocharisrhytidophylla* C.Y. Wu ex H.W. Li (Li 1983; Zhang et al. 2019) (Fig. 3).

## ﻿Materials and methods

Vouchers of the new species were collected from Lancang County, Yunnan Province, China. Photographs and phenology data were obtained during field expeditions. Morphological examinations and measurements of the new species were conducted on both living plants in the wild and herbarium specimens. All morphological characters are described according to the terminology presented by Wang et al. (1990, 1998). The preliminary conservation assessment for the new species was evaluated according to the guidelines of the IUCN Red List Categories and Criteria (IUCN 2019).

## ﻿Taxonomic treatment

### ﻿ Oreocharis polyneura

Taxon classificationPlantaeLamialesGesneriaceae

Y.H.Tan, F.Wen & Y.X.Gong sp. nov.

EE462EFD-104E-57AC-8B96-AEA9E36315F2

urn:lsid:ipni.org:names:77308755-1

Figs 1 , 2

#### Diagnosis.

*Oreocharispolyneura* is similar to *O.rhytidophylla* in having more obvious lateral veins forming crosslinked network on the adaxial surface of leaf blades, but can be distinguished by having more lateral veins (12–15 pairs vs. 7–9 pairs) of leaf blades, shorter corolla tubes (the length ratio of corolla tube to corolla lobes = 1.2–2.9 vs. 3.4–6) and shorter pistils (6–8 mm long vs. 27–30 mm long).

#### Type.

China. Yunnan Province, Puer City, Lancang County, Zhutang Town, on rocks or cliffs in evergreen broad-leaved forests in ravines, 22°45'44.28"N, 99°43'26.81"E, elev. 2137 m, 29 July 2021, *Y.X. Gong G344* (holotype: HITBC0079099; isotypes: HITBC, IBK). Fig. 2.

#### Description.

Perennial herbs. ***Rhizomes*** subterete, short, straight, with numerous fibrous roots. ***Leaves*** basally forming a rosette, 4–8, petiolate; petioles terete, 1.2–7.0 cm long, 3–5 mm in diameter, densely rust-brown pannose, leaf blade thickly chartaceous, elliptic to ovate-elliptic, 4–13 × 2–7 cm, apex rounded, base rounded to subcordate, margin serrate to crenate, adaxially green, glabrous, abaxially whitish-green, densely rust-brown pannose along veins, lateral veins 12–15 on each side of the midrib, adaxially conspicuously sunken, abaxially conspicuously prominent, anastomosed forming on both surfaces. ***Inflorescences*** cymose, axillary, 2–4, 2–4-branched, 4–10-flowered; peduncles 4.5–9.0 cm long, ca. 1–3 mm in diameter, rust-brown lanate; bracts 3, verticillate, each 8.5–9.5 × 3.2–4.2 mm, lanceolate to elliptic, margins usually entire, occasionally 2–3-denticulate, adaxially glabrous, abaxially rust-brown pannose; pedicels 1.0–3.5 cm long, ca. 1 mm in diameter, sparsely puberulent. ***Calyces*** actinomorphic, 5-parted from the base, segments equal, linear-lanceolate, 7.5–9.0 × 1.5–2.5 mm, margin dentate, adaxially glabrous, abaxially rust-brown pannose. ***Corollas*** yellow, 1.8–2.2 cm long, outside glabrous, inside sparsely glandular-puberulent in the throat on adaxial lobes; tube narrowly infundibuliform, slightly bent near the throat, 1–1.5 cm long, 4–5.5 mm in diameter, proximally and distally almost equal in width; limb indistinctively bilabiate, adaxial lip 2-lobed, lobes obovate to elliptic, 5.8–6.2 × 3.8–4.8 mm, apex obtuse, margin entire, abaxial lip 3-lobed, lobes obovate to elliptic, almost equal, 6.2–8.2 × 5.2–6.2 mm, apex rounded, margin entire. ***Stamens*** 4, included, two pairs of stamens cohering at the anther tips, adnate to corolla tube 4–6 mm from base; adaxial stamens 3.5–4.5 mm long, abaxial stamens 5–6 mm long; filaments yellow, linear, glabrous; anthers basifixed, reniform, 2-locular, dehiscing transversely; staminode 1, 1.4 mm long, adnate to corolla tube 1.8 mm from the base. ***Pistils*** 6–8 mm long, glabrous; ovary 5–6.5 mm long, ca. 1.5 mm in diameter, glabrous, with two parietal placentae; style 1–1.5 mm long; stigma 1, disciform, retuse, edge flipped outwardly, 1.5–2.0 mm in diameter. ***Discs*** ring-shaped, ca. 2.3 mm high, margin slightly undulate with 5 irregularly and shallow lobes. ***Capsules*** indistinctively 4-angled to subterete, ca. 4 cm long, ca. 3 mm in diameter, glabrous.

#### Phenology.

Flowering from July to October; fruiting from September to November.

#### Etymology.

Greek *polys*, many, and *neuron*, nerve, alluding to abaxially conspicuous lateral leaf veins

#### Habitat and distribution.

**Figure 4.**
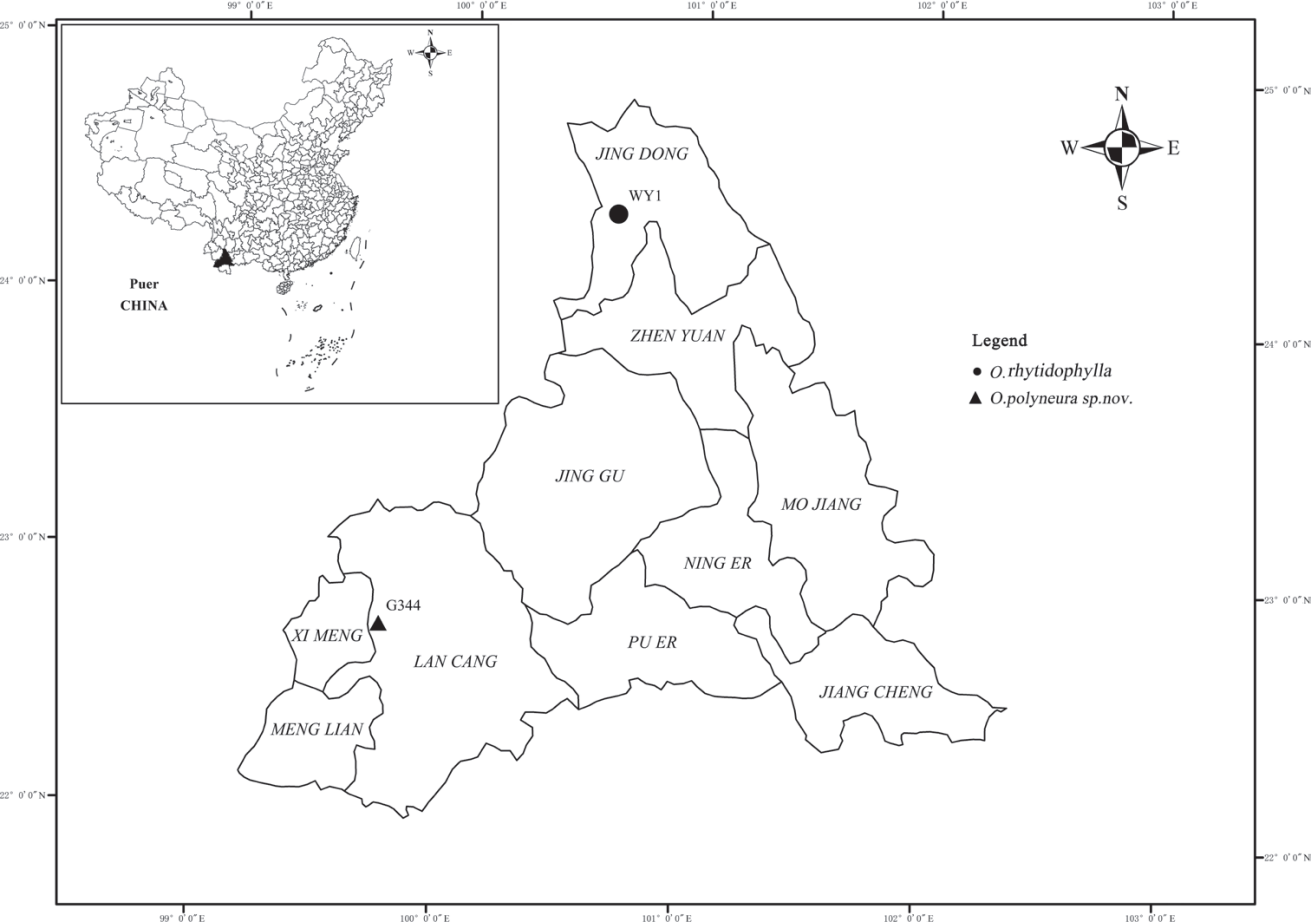
Distribution of *Oreocharispolyneura* (black triangle) and *O.rhytidophylla* (black circle).

Endemic to Zhutang town, Lancang county, Puer city, Yunnan Province, China (Fig. 4). It grows on rocks or cliffs under evergreen broad-leaved forests in ravines at elevations of 1900–2200 m.

#### Conservation status.

*Oreocharispolyneura* is currently known only from its type locality. Based on our present knowledge and available data, its conservation status is assessed as “Data Deficient” (DD; IUCN 2019).

#### Vernacular name.

The Chinese name of the new species is “Duō Mài Mǎ Líng Jù Tái” (多脉马铃苣苔). The first two words, Duō Mài mean the numerous veins of the leaf blades, and the rest four words mean the genus *Oreocharis* in mandarin.

#### Notes.

Morphologically, *Oreocharispolyneura* is most similar to *O.rhytidophylla* in its leaves and inflorescences but is readily distinguishable by an array of characters (see Table 1).

**Table 1.** Morphological comparison between *Oreocharispolyneura* and *O.rhytidophylla*.

Character	*Oreocharispolyneura*	*Oreocharisrhytidophylla*
Petiole	terete	plane
Leaf	lateral veins 12–15 pairs	lateral veins 7–9 pairs
Peduncle	4.5–9 cm long	up to 14 cm long
Corolla	1.8–2.2 cm long; tube narrowly infundibuliform	3.5–3.7 cm long; tube cylindric
Length ratio of corolla tube to corolla lobes	1.2–2.9	3.4–6
Stamen	two pairs cohered at the anther tips, adnate to corolla tube 4–6 mm from base	adaxial stamens cohered in pair, abaxial stamens free, adnate to corolla tube 8–9 mm from base
Pistil	6.0–8.0 mm long; ovary 5.0–6.5 mm long	27–30 mm long; ovary 17–18 mm long

## Supplementary Material

XML Treatment for Oreocharis polyneura
